# Making Judgments of Learning Increases Restudy Choices: Evidence for Reactivity in Metacognitive Control

**DOI:** 10.3390/jintelligence14070145

**Published:** 2026-07-11

**Authors:** Meng Liu, Yanlu Qi, Mingliang Hu, Ningxin Su, Baike Li

**Affiliations:** 1 College of Psychology, Liaoning Normal University, Dalian 116029, China; lium_87@lnnu.edu.cn (M.L.); larsyl@163.com (Y.Q.); 15541679191@163.com (M.H.); 2 Joint Education Institute of Zhejiang Normal University and University of Kansas, Zhejiang Normal University, Jinhua 321004, China; suningxin@zjnu.edu.cn

**Keywords:** JOLs, metacognitive control, reactivity effect, restudy choice

## Abstract

An emerging body of research has shown that requiring participants to make concurrent JOLs during learning can reactively influence memory performance, a phenomenon known as the reactivity effect. However, it remains unclear whether making JOLs can also reactively alter metacognitive control. The present study addressed this question across three types of materials: unrelated word pairs, general knowledge questions, and related word pairs. After studying each item, participants decided whether they would choose to restudy it. In JOL conditions, participants made an item-by-item JOL during learning, while in no-JOL conditions, no such judgment was required. Across all materials, making JOLs reliably increased the proportion of items selected for restudy, indicating a robust reactivity effect on metacognitive control. These findings suggest that JOL reactivity should be considered not only in relation to memory performance but also in relation to metacognitive control.

## 1. Introduction

Effective self-regulated learning depends in part on learners’ ability to evaluate their current state of learning and to use that information to guide subsequent study decisions (Karpicke, 2009; Son & Metcalfe, 2000). In memory and decision-making research, metacognitive judgments such as judgments of learning (JOLs) and confidence ratings (CRs) are often treated as indicators of monitoring, and the relation between such judgments and task performance is commonly used to assess metacognitive ability (Dunlosky & Tauber, 2016; Fleming & Daw, 2017; Fleming & Lau, 2014; Mueller et al., 2014; Mueller & Dunlosky, 2017). However, an important theoretical and methodological issue is whether eliciting such judgments is measurement neutral. If the act of making a metacognitive judgment alters encoding, retrieval, or subsequent control behavior (i.e., decisions about whether and how to restudy or allocate further study time), then metacognitive judgments do not merely reveal the state of monitoring; they may also change the learning process that they are intended to measure.

Over the past decade, however, accumulating evidence has shown that eliciting JOLs is not always measurement neutral. Rather, requiring participants to make JOLs can itself alter ongoing cognitive processing, a phenomenon commonly referred to as the JOL reactivity effect (Ariel et al., 2021; Chang & Brainerd, 2023, 2024; Double et al., 2018, 2025; Double & Birney, 2018, 2019; Halamish, 2018; Halamish & Undorf, 2023; Ingendahl et al., 2025; Ingendahl & Undorf, 2026; Li et al., 2024b; Soderstrom et al., 2015; W. L. Zhao et al., 2022). For example, Soderstrom et al. (2015) found that making JOLs enhanced later memory for related word pairs (i.e., positive reactivity effect), whereas the effect was weaker or absent for weakly related pairs. Subsequent studies have further shown that the direction and magnitude of JOL reactivity are moderated by learning materials, test format (e.g., cued-recall, free recall, and recognition), task context, and individual differences (Ingendahl et al., 2025; Maxwell & Huff, 2022; Murphy et al., 2024; Rivers et al., 2023; Undorf et al., 2024; W. L. Zhao et al., 2022; Zheng et al., 2025). For example, W. Zhao et al. (2025) found that working memory capacity independently and positively predicts JOL reactivity, with this predictive effect remaining significant even after controlling for other cognitive constructs (e.g., attentional control, episodic memory, general fluid intelligence). Myers et al. (2020) found that making JOLs improves recognition memory for word pairs and word lists, although its effect on free recall of words appears to be relatively small. Undorf et al. (2024) further found that for unrelated word pairs, negative JOL reactivity was stronger in controlled settings, such as laboratory contexts, than in uncontrolled settings, such as unsupervised online environments. In the memory literature, the JOL reactivity effect is reliable but not uniform. Positive reactivity has been reported for semantically related word pairs, pictures, and word lists (Li et al., 2024b; Shi et al., 2023; Soderstrom et al., 2015), whereas negative reactivity has been observed for unrelated word pairs (Li et al., 2024b; Mitchum et al., 2016). Null effects have also been reported for materials such as general knowledge questions and text comprehension passages (Ingendahl & Undorf, 2024). Thus, although JOL reactivity is now well-documented, its memorial consequences appear to depend on the nature of the materials and task conditions.

Several theoretical accounts have been proposed to explain why JOLs affect memory differently across materials and paradigms. According to the cue-strengthening hypothesis, making JOLs directs learners’ attention to diagnostic cues, such as cue–target associative strength, thereby improving later memory when the test is sensitive to those cues (Soderstrom et al., 2015). In contrast, the dual-task cost account proposes that making JOLs imposes additional processing demands during encoding and may impair learning, particularly for difficult materials such as unrelated word pairs (Halamish & Undorf, 2023; Li et al., 2024b; Mitchum et al., 2016; Witherby et al., 2023). The changed-goal hypothesis further suggests that making JOLs can make item difficulty more salient and shift learners toward a more efficiency-oriented allocation strategy, such that relatively easier items receive more attention than difficult items (Janes et al., 2018; Mitchum et al., 2016). These accounts have substantially advanced the understanding of why making JOLs can influence subsequent memory. However, they have primarily addressed the effect of JOLs on encoding and later performance. Less is known about whether making JOLs also changes learners’ subsequent control decisions.

Within classic metacognitive frameworks, monitoring is assumed to guide control (Dunlosky & Hertzog, 1998; Metcalfe & Kornell, 2005; Nelson & Narens, 1994; Schaper et al., 2023; Thiede & Dunlosky, 1999). The present study builds on this literature by distinguishing between two forms of JOL reactivity. Memory-level JOL reactivity refers to the effect of making JOLs on later memory performance (e.g., Ingendahl et al., 2025; Mitchum et al., 2016). Control-level JOL reactivity refers to the effect of making JOLs on subsequent learning-control behavior, such as whether an item is selected for restudy. This distinction is theoretically important because the monitoring-control framework concerns not only the accuracy of monitoring, but also how monitoring information is translated into regulation (Metcalfe & Kornell, 2005; Nelson & Narens, 1994; Thiede & Dunlosky, 1999). A JOL may change memory performance by altering encoding, but it may also change the learner’s control policy by altering how evidence of learning, uncertainty, or future forgetting is used to guide restudy decisions (Tauber & Rhodes, 2012). Thus, memory-level and control-level reactivity are conceptually separable. Making JOLs could impair, enhance, or leave memory unchanged while still influencing learners’ decisions about whether further study is needed.

Prior studies provide suggestive but mixed evidence that making JOLs may influence learning control. Mitchum et al. (2016, Experiments 1 and 2) found that study time was negatively related to item difficulty and that making JOLs attenuated this relation, suggesting that JOLs may increase awareness of item difficulty and shift study behavior toward a more efficiency-oriented pattern. However, this pattern has not been consistently replicated (Janes et al., 2018; Li et al., 2024b). Similarly, Ingendahl and Undorf (2025) found that immediate JOLs altered reported strategy use and that these strategy changes statistically mediated negative reactivity effects for some materials, whereas other studies have found no reliable differences between JOL and no-JOL conditions in retrospective strategy reports (Mitchum et al., 2016, Experiment 1; Rivers et al., 2021, Experiment 3). More directly, Tauber and Rhodes (2012, Experiment 5) examined restudy choices across JOL (i.e., the likelihood of recalling each item on a later memory test), JOR (judgments of retention, i.e., how long they would be able to remember each word on a minute scale, 0–60 min), and no-judgment conditions. They found that JOLs did not significantly change the proportion of items selected for restudy relative to a no-judgment condition. This finding provides an important precedent, but it also leaves open whether control-level JOL reactivity may emerge under more sensitive item-level designs or across materials associated with different memory-level reactivity profiles. Thus, existing work does not yet provide a systematic test of whether JOLs reactively alter restudy decisions, or whether such changes merely track the direction of JOL-induced memory effects.

Soliciting JOLs may affect restudy choices; related evidence comes from research on confidence-rating reactivity in perceptual decision-making. Li et al. (2024a) found that requiring participants to make CRs after perceptual decisions increased accuracy, slowed responses, and increased decision thresholds. They argued that making CRs can reactively influence metacognitive control by making uncertainty more salient and encouraging a more conservative decision policy. Although making CRs in perceptual decision-making and JOLs in memory tasks are not identical, they share a common feature: both require participants to explicitly evaluate the quality of their cognitive processing. By analogy, making JOLs during learning may increase the proportion of restudy choice by stimulating learners’ meta-awareness of uncertainty and encouraging a more cautious approach to study regulation. In a learning context, this conservativeness would not be expressed as a higher perceptual decision threshold, but as a higher criterion for deciding that an item has been sufficiently learned.

However, increased restudy choices induced by making JOLs could arise from at least two different mechanisms. One possibility is a compensatory account. If making JOLs disrupts encoding or makes learning feel more difficult, more effortful, less fluent, or less secure, learners may respond by selecting more items for restudy. This interpretation is broadly consistent with discrepancy-reduction accounts of self-regulated study, according to which learners allocate additional study to items that are perceived to fall short of a desired level of mastery (Dunlosky & Hertzog, 1998; Son & Metcalfe, 2000; Thiede & Dunlosky, 1999). From this perspective, increased restudy choices should be most likely when JOLs impair memory. If JOL-induced restudy choices merely compensate for memory-level disruption, then control-level reactivity should primarily occur for materials that show negative memory-level reactivity.

A second possibility is that JOLs alter learners’ control policy in a way that is not fully reducible to memory impairment—a conservative-shift account. The region of proximal learning model proposes that learners preferentially allocate study to items that have not yet been mastered but are still close enough to mastery to benefit from further study (Metcalfe & Kornell, 2005). More generally, monitoring-based control theories propose that study decisions depend not only on the actual state of learning but also on how learners interpret monitoring cues and translate those cues into control decisions (Nelson & Narens, 1994; Thiede & Dunlosky, 1999). From this perspective, making JOLs may direct learners’ attention to uncertainty, future test demands, or the possibility of forgetting, thereby changing the criterion for deciding whether an item requires further study. Drawing on recent work on confidence-rating reactivity in perceptual decision-making, the conservative-shift account proposes that repeatedly requiring metacognitive judgments may heighten learners’ awareness of such uncertainty (Konstantinidis & Shanks, 2014) and shift their control criterion toward a more cautious direction when deciding whether further study is needed (Li et al., 2024a). If this account is correct, making JOLs may increase restudy choices even when memory-level JOL reactivity is negative, null, or positive. Such a pattern would indicate that control-level JOL reactivity is at least partly dissociable from memory-level JOL reactivity.

The present study was designed to test this possibility across three types of materials that have been associated with different memory-level reactivity profiles. Experiment 1 used unrelated word pairs, for which making JOLs was expected to impair later memory. Experiment 2 used general knowledge questions, for which making JOLs was expected to have no reliable effect on memory. Experiment 3 used related word pairs, for which making JOLs was expected to enhance later memory. In each experiment, participants studied items in JOL and no-JOL conditions and then made item-by-item restudy choices. This design allowed us to examine whether eliciting JOLs changes subsequent metacognitive control and whether such changes depend on the direction of memory-level reactivity.

We predicted that making JOLs would increase the proportion of items selected for restudy. More importantly, by comparing materials associated with negative, null, and positive memory-level reactivity, we tested whether restudy-choice reactivity simply follows JOL-induced memory impairment or instead emerges across materials despite different memory outcomes. If increased restudy choices occur only when JOLs impair memory, this would support a compensatory interpretation. By contrast, if JOLs increase restudy choices even when memory is unchanged or improved, this would suggest that making metacognitive judgments can alter learners’ control decisions in a way that is not fully determined by their effects on memory performance. In this way, the present study extends JOL-reactivity research from memory outcomes to metacognitive-control behavior and clarifies how the act of metacognitive monitoring can itself reshape subsequent regulation.

## 2. Experiment 1

Experiment 1 aimed to investigate whether making JOLs would affect the proportion of restudy choices during the learning of unrelated word pairs.

### 2.1. Participants

To determine the required sample size, 10 participants were recruited for a pilot study, following the same procedure as the formal experiment. The pilot study revealed that the effect size (Cohen’s *d*) for the difference in the proportion of restudy choices was 0.54. A power analysis in G*Power 3.1.9.5 (Faul et al., 2007) based on this estimate indicated that 29 participants would be required to detect an effect with 80% power at *α* = 0.05. Because this estimate was slightly below 30, and to reduce the potential instability associated with very small samples, we set a minimum target sample size of 30 participants for the formal experiment. Ultimately, 33 participants were recruited from the Liaoning Normal University (LNNU) participant pool, including 24 women and 9 men (*M* = 21.00 years, *SD* = 1.56). No participants were excluded from Experiment 1. Each participant provided informed consent, was individually tested in a soundproof cubicle, and received financial compensation.

### 2.2. Material

Seventy Chinese word pairs were selected from Hu et al. (2016), in which participants rated the semantic relatedness of each pair on a scale from 1 (completely unrelated) to 4 (strongly related). The average relatedness rating for the selected pairs was 1.374 (*SD* = 0.339), indicating that they were semantically unrelated. Six pairs were used for practice, while the remaining 64 pairs were included in the formal experiment. To minimize item-selection effects, the 64 pairs were randomly divided into four lists of 16 pairs, which were then randomly assigned to the two conditions (JOL vs. no-JOL). The order of blocks and the sequence of pairs within each block were randomized by the computer for each participant. All stimuli were presented using the Matlab 2023b and the Psychtoolbox package 3.0.19 (Kleiner et al., 2007).

### 2.3. Design and Procedure

The experiment involved a within-subject design (JOL vs. no-JOL). Before the formal experiment, participants completed a practice task to familiarize themselves with the experimental learning procedure. The practice task followed the same procedure as the formal experiment. In the formal experiment, participants studied four blocks of word pairs, with 16 pairs in each block. In two blocks (JOL condition), participants were instructed to make an item-by-item JOL during the learning process, whereas in the other two blocks, they were not required to do so (no-JOL condition).

**Figure 1:**
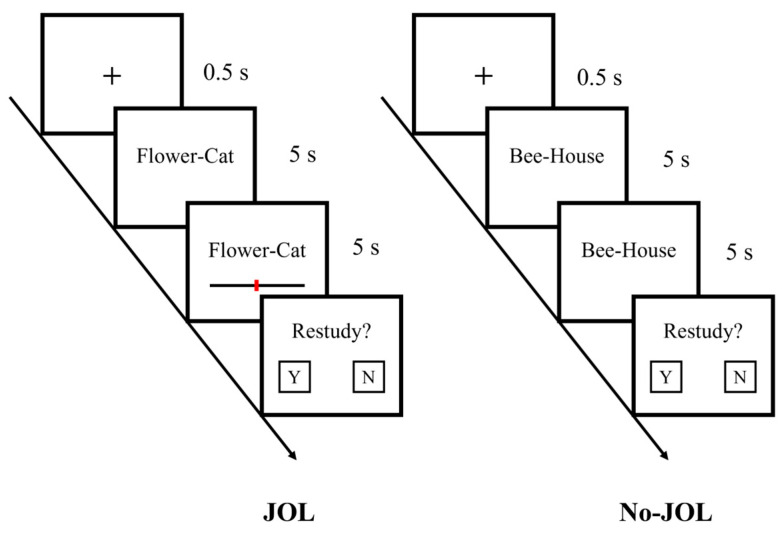
Flow chart depicting the study procedure in Experiment 1.

The study procedure is illustrated in Figure 1. In the no-JOL condition, participants studied the word pairs sequentially. Before each word pair was shown, a cross (“+”) appeared at the center of the screen for 0.5 s as an inter-stimulus interval. Following this, a word pair (e.g., “Flower–Cat”) was displayed on the screen for 10 s. Afterward, the prompt “Restudy?” appeared, asking participants whether they wanted to restudy the pair before the test. Participants clicked “Yes” or “No” on the screen. This process was repeated until all 16 pairs in the block had been studied.

The procedure for the JOL condition was similar to that for the no-JOL condition, except that participant needed to make a JOL after learning each word pair. Specifically, each word pair was displayed for 10 s: for the first 5 s, the pair was presented alone, and for the next 5 s, a slider appeared below it. Participants used the slider to predict the likelihood of remembering the pair on a later memory test, on a scale ranging from 0 (“Sure I will not remember it”) to 100 (“Sure I will remember it”). If participants successfully made a JOL within the 5 s window, the word pair remained on the screen for the remaining duration. If not, a message box appeared reminding them to complete the task within the required time on subsequent trials. Participants clicked the mouse to dismiss the message and proceed to the next trial^1^.

After studying all word pairs, participants completed a 5 min distractor task involving math problems (e.g., 7 + 45 = ___?). Then, they were informed that the items they had chosen to restudy during the learning phase would not actually be restudied. Following this, a cued recall test was conducted for all 64 word pairs. Cue words were presented one by one in random order, and participants were instructed to recall the corresponding target words. There was no time limit or feedback during the recall test.

### 2.4. Results and Discussion

**Figure 2:**
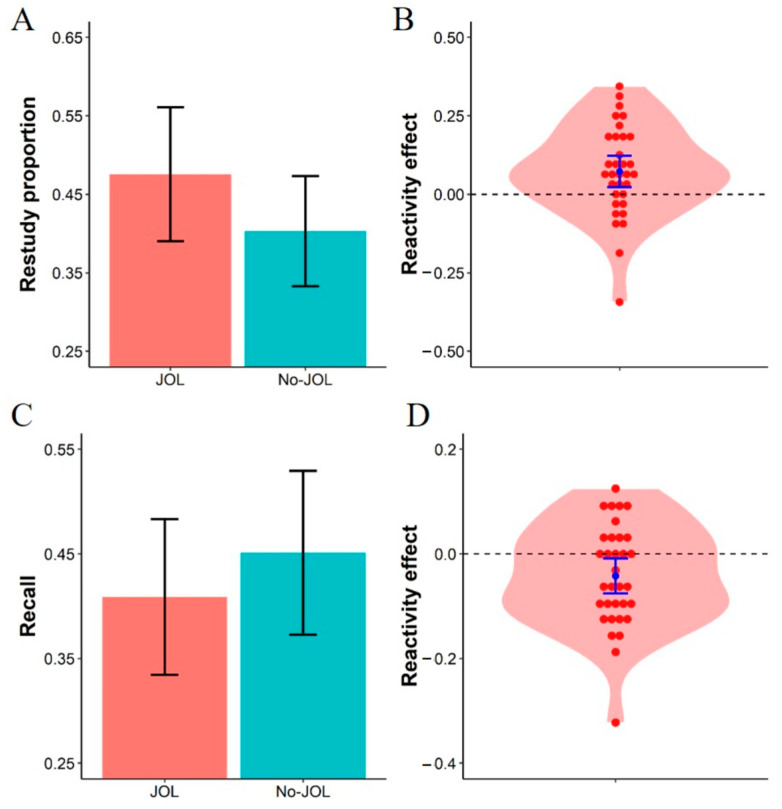
**Restudy choices and cued recall performance in Experiment 1.** (Panel (**A**)): Proportion of restudy choices in the JOL and no-JOL conditions. (Panel (**B**)): Violin plot displaying the distribution of reactivity effects on restudy choices (i.e., the difference between the proportion of restudy choices in the JOL and no-JOL conditions). (Panel (**C**)): Cued recall performance for JOL and no-JOL word pairs. (Panel (**D**)): Violin plot illustrating the distribution of reactivity effects (i.e., the difference between cued recall for JOL word pairs and cued recall for no-JOL word pairs). In Panels (**A**,**C**), bars represent condition-level means, and error bars indicate 95% confidence intervals around those means. In Panels (**B**,**D**), each red dot represents the reactivity effect score for an individual participant, and the blue dots represent the group mean reactivity effect. Error bars indicate the 95% confidence intervals (CIs) around the mean reactivity effect. The dashed horizontal line in the violin plots indicates zero reactivity.

The primary analyses examined whether making JOLs affected restudy choices and cued recall performance. The proportions of restudy choices and recall performance are shown in Figure 2. Along with frequentist analyses, the two-sided Bayesian paired t-tests were conducted to evaluate whether the observed data favored the null hypothesis (*H*_0_: absence of the reactivity effect) over the alternative hypothesis (*H*_1_: presence of the reactivity effect). The Bayes factor (*BF*_10_) quantifies the strength of evidence in favor of the alternative hypothesis relative to the null. Specifically, a *BF*_10_ greater than 3 indicates substantial evidence supporting the alternative hypothesis, whereas a *BF*_10_ less than 0.33 indicates substantial evidence favoring the null hypothesis (Barchard, 2015; Mulder & Wagenmakers, 2016). All Bayesian analyses reported here were performed using JASP version 0.18.1.0 (http://jasp-stats.org/, accessed on 1 July 2025), with default parameter settings. Unless otherwise noted, all tests were two-sided. For clarity, positive memory reactivity refers to better recall performance in the JOL condition than in the no-JOL condition, whereas negative memory reactivity refers to the reverse pattern (Ingendahl et al., 2025). In parallel, positive restudy-choice reactivity refers to a higher proportion of restudy choices in the JOL condition than in the no-JOL condition, whereas negative restudy-choice reactivity refers to the reverse pattern.

For restudy choices, participants selected a greater proportion of items for restudy in the JOL condition (*M* = 0.48, *SD* = 0.25) than in the no-JOL condition (*M* = 0.40, *SD* = 0.21), mean difference = 0.07, 95% CI [0.02, 0.13], *t*(32) = 2.86, *p* = .007, *d*z = 0.50, *BF*_10_ = 5.60 (see Figure 2A,B). For descriptive purposes, 23 participants showed positive restudy-choice reactivity, eight showed negative restudy-choice reactivity, and two showed no difference between conditions. For cued recall performance, participants recalled fewer targets in the JOL condition (*M* = 0.41, *SD* = 0.22) than in the no-JOL condition (*M* = 0.45, *SD* = 0.23), mean difference = −0.04, 95% CI [−0.08, −0.01], *t*(32) = −2.46, *p* = .020, *d*z = −0.43, *BF*_10_ = 2.49 (see Figure 2C, D). For descriptive purposes, 18 participants showed negative memory reactivity, five showed no difference, and 10 showed positive memory reactivity. Thus, Experiment 1 was consistent with prior findings showing negative memory reactivity for unrelated word pairs (Li et al., 2024b; Mitchum et al., 2016), while also showing that making JOLs increased the proportion of items selected for restudy.

Additional analyses were conducted to characterize the relation among JOLs, monitoring accuracy, and restudy choices. Participants successfully provided item-by-item JOLs for nearly all trials in the JOL condition (*M* = 99.90%, *SD* = 0.06%). The mean JOL rating was 52.74 (*SD* = 12.71). To assess whether JOLs reflected subsequent memory performance, we calculated a within-participant gamma correlation between JOLs and recall accuracy. The average gamma correlation was significantly greater than zero, *G* = 0.23, *SD* = 0.29, 95% CI [0.12, 0.33], *t*(32) = 4.43, *p* < .001, *d* = 0.77, indicating that participants showed above-chance relative monitoring accuracy. The within-participant gamma correlation between JOLs and restudy choices was significantly below zero, *G* = −0.56, *SD* = 0.24, 95% CI [−0.72, −0.44], *t*(32) = −7.38, *p* < .001, *d* = −1.28, indicating that participants were more likely to select items for restudy when they had assigned lower JOLs to those items. These results suggest that restudy choices in the JOL condition were systematically guided by learners’ subjective monitoring of item difficulty.

Finally, to examine whether making JOLs changed the alignment between restudy choices and subsequent recall accuracy, the tie-corrected gamma correlations between restudy choices and recall accuracy were separately calculated for the JOL and no-JOL conditions. This analysis was conducted at the participant level and required variability in restudy choices within each condition. Ten participants could not be included in this gamma-correlation analysis because their restudy choices showed insufficient variability, leaving 23 participants for this analysis. This exclusion applied only to the gamma-correlation analysis and not to the primary analyses reported above. Mean gamma was −0.31 (*SD* = 0.42) in the JOL condition and −0.34 (*SD* = 0.34) in the no-JOL condition. After Fisher’s *z* transformation, the two gamma coefficients did not differ significantly, *t*(22) = −0.39, *p* = .70, *d*z = −0.08. Thus, in both conditions, restudy choices tended to be directed toward items that were less likely to be recalled, but Experiment 1 did not provide evidence that making JOLs strengthened this restudy–accuracy association.

Taken together, Experiment 1 showed that making JOLs increased restudy choices while reducing cued recall performance for unrelated word pairs. Because increased restudy choices co-occurred with impaired memory performance, Experiment 1 alone cannot determine whether the increase in restudy choices reflects a direct control-level reactivity effect or a compensatory response to JOL-related disruption of learning. Experiments 2 and 3 therefore tested whether increased restudy choices would still emerge when JOL-induced memory impairment was absent or reversed.

## 3. Experiment 2

Experiment 2 aimed to examine whether making JOLs would increase restudy choices during the learning of general knowledge questions. A second aim was to test whether the increase in restudy choices observed in Experiment 1 could be explained solely as a compensatory response to JOL-related memory impairment. Specifically, making JOLs might disrupt encoding or increase the perceived difficulty of learning (Mitchum et al., 2016), leading participants to experience the items as less securely learned and therefore to select more items for restudy.

General knowledge questions were well-suited for testing this possibility because prior research has shown that making JOLs does not reliably affect memory performance for this type of material (Ingendahl & Undorf, 2024). If increased restudy choices merely reflect compensation for impaired learning, then the effect should be reduced or absent when JOLs are less likely to impair memory. By contrast, if making JOLs continues to increase restudy choices for general knowledge questions, this would provide evidence that restudy-choice reactivity is not reducible to JOL-induced memory impairment alone.

### 3.1. Participants

To determine the required sample size, eight participants were recruited for a pilot study, following the same procedure as the formal experiment. The pilot study revealed that the effect size (*d*) for the difference in the proportion of restudy choices was 0.610. A power analysis conducted using G*Power 3.1.9.5 (Faul et al., 2007) indicated that 24 participants were needed to detect a significant effect on restudy choices, with power set at 0.80 and *α* = 0.05. Because this estimate was slightly below 30, and to reduce the potential instability associated with very small samples, we set a minimum target sample size of 30 participants for the formal experiment. Ultimately, 37 participants were recruited from LNNU’s participant pool, including 33 women and 4 men (*M* = 22.19 years, *SD* = 2.04). No participants were excluded from Experiment 2. Each participant provided informed consent, was individually tested in a soundproof cubicle, and received financial compensation.

### 3.2. Material

Three hundred general knowledge questions (e.g., “What is the name of the city that successfully hosted the Asian Games in 2010?—Guangzhou”) were selected from online knowledge contest question banks, covering topics such as art and architecture, health, entertainment, cuisine, geography, history, literature, science and nature, sports, and technology. A total of 892 participants were recruited to assess the difficulty of these questions. From this pool, 70 questions were chosen as experimental materials, representing all 10 topics, with seven questions per topic. The average difficulty of the selected questions was 0.178 (*SD* = 0.054, range: 0.057–0.273). Six questions were used for practice, while the remaining 64 were used in the formal experiment. To minimize item-selection effects, the 64 questions were randomly divided into four lists of 16 questions and randomly assigned to the two conditions (JOL vs. no-JOL). Additionally, the block order and question order within each block were randomized by the computer for each participant. All stimuli were presented using PsychoPy 2024.2.4 software (Peirce et al., 2019).

### 3.3. Design and Procedure

Similar to Experiment 1, Experiment 2 used a within-subject design (JOL vs. no-JOL). The procedure for Experiment 2 was also similar to that of Experiment 1, with one key difference: participants studied 64 general knowledge questions. Each question was presented for 10 s, after which participants decided whether they wanted to restudy the item before the test. The 64 questions were divided into four lists, with 16 questions in each list. Two lists were randomly assigned to the JOL condition, while the other two lists were assigned to the no-JOL condition. In the JOL condition, participants made JOLs using a slider during the learning phase. In the no-JOL condition, no such JOLs were made. After completing the learning phase, participants were informed that the items they had chosen to restudy would not actually be restudied. They then completed a 5 min distractor task, followed by a cued recall test.

### 3.4. Results and Discussion

**Figure 3:**
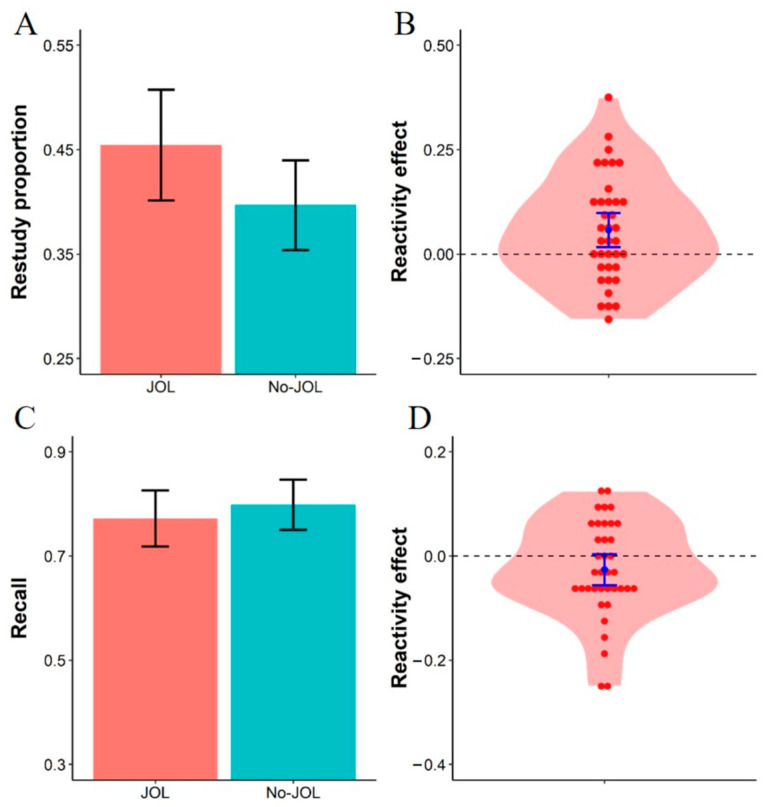
**Restudy choices and cued recall performance in Experiment 2.** (Panel (**A**)): Proportion of restudy choices in the JOL and no-JOL conditions. (Panel (**B**)): Violin plot displaying the distribution of reactivity effects on restudy choices (i.e., the difference between the proportion of restudy choices in the JOL and no-JOL conditions). (Panel (**C**)): Cued recall performance for JOL and no-JOL general knowledge questions. (Panel (**D**)): Violin plot illustrating the distribution of reactivity effects (i.e., the difference between cued recall for JOL and no-JOL general knowledge questions). In Panels (**A**,**C**), bars represent condition-level means, and error bars indicate 95% confidence intervals around those means. In Panels (**B**,**D**), each red dot represents the reactivity effect score for an individual participant, and the blue dots represent the group mean reactivity effect. Error bars indicate the 95% confidence intervals (CIs) around the mean reactivity effect. The dashed horizontal line in the violin plots indicates zero reactivity.

Test performance data were analyzed using the same methods as in Experiment 1. For restudy choices, participants selected a greater proportion of items for restudy in the JOL condition (*M* = 0.45, *SD* = 0.17) than in the no-JOL condition (*M* = 0.40, *SD* = 0.13), mean difference = 0.06 [0.02, 0.10], *t*(36) = 2.75, *p* = .009, *d*z = 0.45, *BF*_10_ = 4.47 (see Figure 3A,B). For descriptive purposes, 22 participants showed positive restudy-choice reactivity, 10 showed negative restudy-choice reactivity, and five showed no difference between conditions. Recall performance was numerically lower in the JOL condition (*M* = 0.77, *SD* = 0.17) than in the no-JOL condition (*M* = 0.80, *SD* = 0.15), but this difference was not statistically significant, mean difference = −0.03 [−0.06, 0.01], *t*(36) = −1.70, *p* = .098, *d*z = −0.28, *BF*_10_ = 0.65 (see Figure 3C,D). For descriptive purposes, 21 participants showed negative memory reactivity, three showed no difference, and 13 showed positive memory reactivity. Thus, Experiment 2 was consistent with prior findings showing no reliable memory reactivity for general knowledge questions (Ingendahl & Undorf, 2024), while also showing that making JOLs increased the proportion of items selected for restudy.

Additional analyses were conducted to characterize the relation among JOLs, monitoring accuracy, and restudy choices. Participants successfully provided item-by-item JOLs for nearly all trials in the JOL condition (*M* = 98.60%, *SD* = 3.00%). The mean JOL rating was 58.91 (*SD* = 11.04). To assess whether JOLs reflected subsequent memory performance, we calculated a within-participant gamma correlation between JOLs and recall accuracy. The average gamma correlation was significantly greater than zero, *G* = 0.33, *SD* = 0.26, 95% CI [0.24, 0.41], *t*(36) = 7.66, *p* < .001, *d* = 1.26, indicating that participants showed above-chance relative monitoring accuracy. The within-participant gamma correlation between JOLs and restudy choices was significantly below zero, *G* = −0.83, *SD* = 0.20, 95% CI [−0.89, −0.76], *t*(36) = −25.30, *p* < .001, *d* = −4.16, indicating that participants were more likely to select items for restudy when they had assigned lower JOLs to those items. These results suggest that restudy choices in the JOL condition were systematically guided by learners’ subjective monitoring of item difficulty.

Finally, to examine whether making JOLs changed the alignment between restudy choices and subsequent recall accuracy, the tie-corrected gamma correlations between restudy choices and recall accuracy were computed separately for the JOL and no-JOL conditions. Fifteen participants could not be included in this gamma-correlation analysis because their restudy choices showed insufficient variability, leaving 22 participants for this analysis. This exclusion applied only to the gamma-correlation analysis and not to the primary analyses reported above. Mean gamma was −0.39 (*SD* = 0.36) in the JOL condition and −0.36 (*SD* = 0.39) in the no-JOL condition. After Fisher’s z transformation, the two gamma coefficients did not differ significantly, *t*(21) = 0.27, *p* = .79, *d*z = 0.06. Thus, in both conditions, restudy choices tended to be directed toward items that were less likely to be recalled, but Experiment 2 did not provide evidence that making JOLs strengthened this restudy–accuracy association.

Experiment 2, using general knowledge questions, replicated the key finding that making JOLs increased the proportion of items selected for restudy. Importantly, this increase occurred even though making JOLs did not reliably impair recall performance for these materials. Thus, the restudy-choice effect observed in Experiment 2 is unlikely to be explained solely as a compensatory response to statistically reliable JOL-related memory impairment. However, because recall performance was numerically lower in the JOL condition, Experiment 2 alone does not fully rule out the possibility that learners experienced JOL trials as somewhat less secure or more effortful.

## 4. Experiment 3

Although Experiment 2 did not reveal a reliable impairment of memory performance for general knowledge questions, recall in the JOL condition was numerically lower than in the no-JOL condition (*d*z = −0.280). Thus, it remains possible that learners experienced JOL trials as somewhat more effortful or less secure, which may still have contributed to increased restudy choices. Experiment 3 was designed to provide a stronger test of this compensatory interpretation by using related word pairs, for which prior research has reported positive JOL reactivity on memory. If increased restudy choices merely reflect compensation for learning disruption, such an effect should be attenuated or absent when making JOLs enhances memory performance. By contrast, if making JOLs still increases restudy choices under these conditions, this would provide stronger evidence that restudy-choice reactivity is not reducible to JOL-related memory impairment. Moreover, Experiment 3 examined whether making JOLs would affect the proportion of restudy choices during the learning of related pairs.

### 4.1. Participants

To determine the required sample size, 11 participants were recruited for a pilot study, which followed the same procedure as the formal experiment. The pilot study revealed that the *d*z for the difference in recall scores between the JOL and no-JOL conditions was 0.32, whereas the effect size for the difference in the proportion of restudy choices was 0.427. A power analysis conducted using G*Power 3.1.9.5 (Faul et al., 2007) indicated that 79 participants were needed to detect a significant reactivity effect on recall, and 43 participants were required to detect a significant effect on restudy choices, with power set at 0.80 and *α* = 0.05. Ultimately, 83 participants were recruited from the participant pool of LNNU, including 66 women and 17 men (*M* = 21.13 years, *SD* = 2.29). To ensure that the final analyzable sample would not fall below the larger target sample size required for detecting the memory-reactivity effect, we recruited slightly more participants than this estimate. No participants were excluded from Experiment 3. Each participant provided informed consent, was individually tested in a soundproof cubicle, and received financial compensation.

### 4.2. Material

Ninety semantically related Chinese word pairs were selected from Hu et al. (2016), where participants rated the semantic relatedness of the pairs on a scale from 1 (completely unrelated) to 4 (strongly related). The average relatedness rating for the selected pairs was 3.46 (*SD* = 0.17). Ten pairs were used for practice, while the remaining 80 pairs were included in the formal experiment. To minimize item-selection effects, the 80 pairs were randomly divided into four lists of 20 pairs each, which were then randomly assigned to the two conditions (JOL vs. no-JOL). Additionally, the order of blocks and the sequence of pairs within each block were randomized by the computer for each participant. All stimuli were presented using the Matlab 2023b and the Psychtoolbox package (Kleiner et al., 2007).

### 4.3. Design and Procedure

Experiment 3 employed a within-subject design (JOL vs. no-JOL). The procedure for Experiment 3 was also largely identical to that of Experiment 1, with two key differences: participants studied 80 related word pairs, and each pair was presented for 8 s. Specifically, the 80 pairs used in the formal experiment were divided into four lists, with 20 related word pairs in each list. Two lists were randomly assigned to the JOL condition, whereas the other two lists were assigned to the no-JOL condition. In the JOL condition, participants made JOLs using a slider during the learning phase. In the no-JOL condition, no such JOLs were made. After completing the learning phase, participants were informed that the items they had chosen to restudy would not actually be restudied. They then completed a 5 min distractor task, followed by a cued recall test.

### 4.4. Results and Discussion

**Figure 4:**
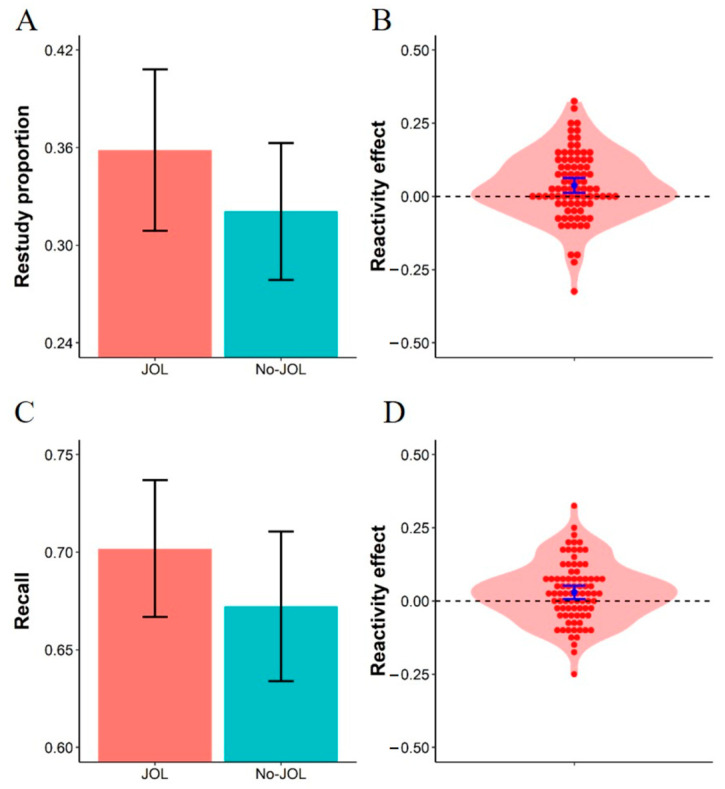
**Restudy choices and cued recall performance in Experiment 3.** (Panel (**A**)): Proportion of restudy choices in the JOL and no-JOL conditions. (Panel (**B**)): Violin plot displaying the distribution of reactivity effects on restudy choices (i.e., the difference between the proportion of restudy choices in the JOL and no-JOL conditions). (Panel (**C**)): Cued recall performance for JOL and no-JOL word pairs. (Panel (**D**)): Violin plot illustrating the distribution of reactivity effects (i.e., the difference between cued recall for JOL word pairs and cued recall for no-JOL word pairs). In Panels (**A**,**C**), bars represent condition-level means, and error bars indicate 95% confidence intervals around those means. In Panels (**B**,**D**), each red dot represents the reactivity effect score for an individual participant, and the blue dots represent the group mean reactivity effect. Error bars indicate the 95% confidence intervals (CIs) around the mean reactivity effect. The dashed horizontal line in the violin plots indicates zero reactivity.

Test performance and restudy-choice data were analyzed using the same methods as in Experiment 1. For restudy choices, participants selected a greater proportion of items for restudy in the JOL condition (*M* = 0.36, *SD* = 0.23) than in the no-JOL condition (*M* = 0.32, *SD* = 0.20), mean difference = 0.04 [0.01, 0.06], *t*(82) = 2.96, *p* = .004, *d*z = 0.32, *BF*_10_ = 6.78 (see Figure 4A,B). For descriptive purposes, 45 participants showed positive restudy-choice reactivity, 24 showed negative restudy-choice reactivity, and 14 showed no difference between conditions. For recall performance, participants recalled more in the JOL condition (*M* = 0.70, *SD* = 0.16) than in the no-JOL condition (*M* = 0.67, *SD* = 0.18), mean difference = 0.03 [0.01, 0.05], *t*(82) = 2.60, *p* = .011, *d*z = 0.29, *BF*_10_ = 2.81 (see Figure 4C,D). For descriptive purposes, 47 participants showed positive memory reactivity, 28 showed negative memory reactivity, and eight showed no difference. Thus, Experiment 3 was consistent with prior findings showing positive memory reactivity for related word pairs, while also showing that making JOLs increased the proportion of items selected for restudy.

Additional analyses were conducted to characterize the relation among JOLs, monitoring accuracy, and restudy choices. Participants successfully provided item-by-item JOLs for nearly all trials in the JOL condition (*M* = 97.71%, *SD* = 2.36%). The mean JOL rating was 66.17 (*SD* = 20.07). To assess whether JOLs reflected subsequent memory performance, we calculated a within-participant gamma correlation between JOLs and recall accuracy. The average gamma correlation was significantly greater than zero, *G* = 0.26, *SD* = 0.26, 95% CI [0.21, 0.31], *t*(82) = 9.53, *p* < .001, *d* = 1.05, indicating that participants showed above-chance relative monitoring accuracy.

We also calculated the within-participant gamma correlation between JOLs and restudy choices. Ten participants could not be included in this gamma-correlation analysis because their restudy choices showed insufficient variability, leaving 73 participants for this analysis. This exclusion applied only to the gamma-correlation analysis and not to the primary analyses reported above. The gamma correlation between JOLs and restudy choices was significantly below zero, *G* = −0.67, *SD* = 0.24, 95% CI [−0.74, −0.63], *t*(72) = −24.19, *p* < .001, *d* = −2.83, indicating that participants were more likely to select items for restudy when they had assigned lower JOLs to those items. These results suggest that restudy choices in the JOL condition were systematically guided by learners’ subjective monitoring of item difficulty.

Finally, to examine whether making JOLs changed the alignment between restudy choices and subsequent recall accuracy, the tie-corrected gamma correlations between restudy choices and recall accuracy separately for the JOL and no-JOL conditions were computed. This analysis was conducted at the participant level and required variability in restudy choices within each condition. Eighteen participants could not be included in this gamma-correlation analysis because their restudy choices showed insufficient variability, leaving 65 participants for this analysis. This exclusion applied only to the gamma-correlation analysis and not to the primary analyses reported above. Mean gamma was −0.66 (*SD* = 0.22) in the JOL condition and −0.30 (*SD* = 0.44) in the no-JOL condition. After Fisher’s z transformation, the two gamma coefficients differed significantly, *t*(64) = 7.17, *p* < .001, *d*z = 0.89. Thus, although restudy choices in both conditions tended to be directed toward items that were less likely to be recalled, this restudy–accuracy association was stronger in the JOL condition than in the no-JOL condition. This finding suggests that, for related word pairs, making JOLs strengthened the alignment between restudy decisions and later item-level recall outcomes.

Experiment 3 showed that making JOLs improved recall performance for related word pairs, consistent with prior findings of positive memory reactivity for this type of material. More importantly, as in Experiments 1 and 2, making JOLs also increased the proportion of items selected for restudy. Because this increase occurred even when JOLs enhanced, rather than impaired, memory performance, the restudy-choice effect is unlikely to be explained solely as a compensatory response to JOL-related disruption of learning.

## 5. Mini Meta-Analytic Summary Across Experiments 1–3

To examine whether the positive effect of making JOLs on restudy choices was stable across materials, a mini meta-analysis was conducted across Experiments 1–3. The target effect in each experiment was the within-subject difference between the JOL and no-JOL conditions in the proportion of restudy choice. Within-subject standardized effect sizes (*d*z) were calculated from the paired-samples comparisons reported for each experiment. The three effect sizes were combined using an inverse-variance fixed-effect model. Because only three experiments were available, the fixed-effect model was treated as the primary analysis, and heterogeneity was quantified using Cochran’s *Q* and *I*^2^. To complement the standardized synthesis, a second analysis combined the raw mean differences in restudy-choice proportions (JOL − no-JOL), thereby providing an estimate of the average absolute increase in the proportion of items selected for restudy when JOLs were elicited.

**Figure 5:**
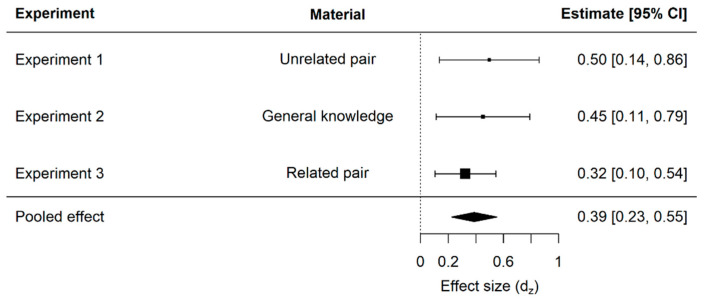
Forest plot of the effect of making JOLs on restudy choice across Experiments 1–3. Effect sizes are presented as Cohen’s *d*z, with horizontal lines indicating 95% confidence intervals (CIs). The pooled effect is shown by the diamond at the bottom of the figure.

The mini meta-analysis showed that the pooled within-subject standardized effect was positive and statistically reliable, *d*z = 0.39, 95% CI [0.23, 0.55], *z* = 4.65, *p* < .001 (see Figure 5). There was no detectable heterogeneity across experiments, *Q*(2) = 0.81, *p* = .67, *I*^2^ = 0%, indicating that the positive effect of making JOLs on restudy choices was relatively stable across the three material types examined here. A complementary synthesis of the raw mean differences showed a pooled increase of 0.048, 95% CI [0.028, 0.068], indicating that eliciting JOLs increased the proportion of items selected for restudy by about 4.8% on average. This pattern is broadly consistent with the possibility that control-level reactivity was more stable across materials than memory-level reactivity.

## 6. General Discussion

The present study examined whether making JOLs reactively alters metacognitive control, as indexed by learners’ item-level restudy choices. Across three experiments, participants decided after studying each item whether they would choose to restudy it. For some items, they made an item-by-item JOL during learning, whereas for other items, they did not. The results showed that making JOLs increased the proportion of items selected for restudy. Importantly, this effect emerged across three types of materials that produced different patterns of memory-level reactivity. Making JOLs impaired later memory for unrelated word pairs in Experiment 1, had no reliable effect on memory for general knowledge questions in Experiment 2, and enhanced memory for related word pairs in Experiment 3. A mini meta-analytic summary across experiments further confirmed a reliable pooled within-subject effect of JOLs on restudy choices. Additional item-level analyses further showed that participants’ JOLs were meaningfully related to subsequent memory performance and restudy choices. Across experiments, JOLs generally showed above-chance relative monitoring accuracy, and lower JOLs were associated with a higher likelihood of selecting an item for restudy. These findings indicate that restudy choices in the JOL condition were not arbitrary, but were systematically related to learners’ subjective monitoring of item difficulty or learning status.

The current study’s findings make two main contributions. First, they extend research on JOL reactivity beyond memory performance. Prior research has primarily examined whether making JOLs changes later memory and has shown that such memory-level reactivity varies substantially across materials and task conditions (Double et al., 2018; Ingendahl et al., 2025; Mitchum et al., 2016; Soderstrom et al., 2015). The present findings suggest that making JOLs can also reactively affect subsequent learning-control decisions. Second, the results suggest that memory-level and control-level JOL reactivity are at least partly dissociable. Although the effect of JOLs on memory differed across Experiments 1–3, the effect of JOLs on restudy choices was consistently positive. Thus, the consequences of making JOLs cannot be fully characterized by their effects on memory performance alone.

### 6.1. Potential Mechanisms for JOL-Induced Increases in Restudy Choices

The present discussion focuses on control-level mechanisms. Memory-level accounts such as the cue-strengthening hypothesis, dual-task cost account, and changed-goal hypothesis are not revisited here, as they primarily address JOL reactivity in encoding rather than control decisions.

One possible explanation for the increase in restudy choices is a compensatory account. This account is broadly consistent with discrepancy-reduction views of self-regulated learning, according to which learners allocate additional study to materials that are perceived to fall short of a desired level of mastery. (Dunlosky & Hertzog, 1998; Son & Metcalfe, 2000; Thiede & Dunlosky, 1999). If learners experience JOL trials as more difficult or less well-learned, they may compensate by selecting more items for restudy.

Experiment 1’s findings are compatible with this possibility. In that experiment, participants studied unrelated word pairs, a type of material for which making JOLs has been shown to produce negative memory-level reactivity, presumably because the additional requirement to monitor learning places demands on limited cognitive resources during a difficult encoding task (Li et al., 2024b; Mitchum et al., 2016). Consistent with this view, making JOLs impaired later memory for unrelated word pairs and increased the proportion of items selected for restudy. This pattern could reflect compensation: learners may have detected, either explicitly or implicitly, that JOL trials were less securely learned and therefore selected more of them for further study. Consistent with the idea that restudy choices reflected perceived learning needs, the item-level analyses showed that items selected for restudy were generally less likely to be recalled later than items not selected for restudy. This pattern indicates that learners’ restudy decisions were sensitive to item difficulty or learning status.

However, the compensatory account cannot fully explain the overall pattern of findings. If increased restudy choices merely reflected compensation for JOL-induced memory impairment, then the effect should have been limited to, or at least most pronounced for, materials showing negative memory-level reactivity. This was not the pattern observed. In Experiment 2, making JOLs increased restudy choices even though it had no reliable effect on memory for general knowledge questions. More strikingly, in Experiment 3, making JOLs also increased restudy choices even though it enhanced later memory for related word pairs, a pattern consistent with prior evidence for positive JOL reactivity in related-pair learning (Soderstrom et al., 2015; Witherby & Tauber, 2017). Thus, the increase in restudy choices did not simply track the direction of JOL-induced changes in memory. These findings suggest that compensation may account for part of the effect under some conditions, especially when making JOLs impairs memory or increases the perceived difficulty of learning.

A second explanation is a conservatism shift account. This account proposes that repeatedly requiring metacognitive judgments heightens learners’ awareness of uncertainty and shifts their decision criterion toward greater caution when deciding whether further study is needed (Li et al., 2024a). In the present context, making JOLs may lead learners to require stronger subjective evidence that an item has been sufficiently learned before declining restudy, thereby increasing the proportion of items selected for restudy across all material types. The cross-material consistency of the effect is broadly consistent with this account.

However, this interpretation requires caution. If making JOLs induced a general conservative shift, one might expect JOLs to reliably alter the relation between restudy decisions and later memory performance, for example, by changing the criterion for declining restudy. The present data did not provide consistent evidence for this pattern. Across experiments, items selected for restudy were generally less likely to be recalled than items not selected, indicating that restudy decisions were sensitive to item difficulty or perceived learning status. However, making JOLs did not consistently change the relation between restudy choices and later recall accuracy across experiments (see Appendix A). Therefore, although a conservative-shift account remains plausible, the present findings do not provide definitive support for this specific mechanism, and alternative explanations cannot be ruled out.

A third related possibility, which may reflect one specific route through which a conservatism shift could arise, is that making JOLs increases the salience of future forgetting, which encourages the learner to adopt a more cautious restudy policy. For example, Tauber and Rhodes (2012) found that a judgment-of-retention (JOR) condition produced a higher proportion of restudy choices than a no-judgment condition, whereas the difference between JOL and no-JOL conditions was not significant. They suggested that JORs, which require participants to predict how long they will remember an item, make the prospect of forgetting especially salient, thereby encouraging a more cautious restudy policy. The negative association between JOL magnitude and restudy choices observed in the present experiments is consistent with this possibility: items judged as less likely to be remembered were more likely to be selected for further study. Therefore, making JOLs might influence the proportion of restudy choices by triggering unconscious processes that predict future forgetting.

The absence of a significant JOL effect in their study may reflect differences in experimental design. Tauber and Rhodes used a between-subjects manipulation, which may be less sensitive to reactivity effects than the within-subject design employed here (Rivers et al., 2021; Greenwald, 1976). In within-subject designs, the contrast between judgment and no-judgment trials may sharpen participants’ awareness of how making judgments influences their learning experience, making reactivity effects easier to detect. This design difference may help explain why the present study observed a significant JOL effect on restudy choices, whereas Tauber and Rhodes did not. Nevertheless, their findings raise the possibility that heightened salience of future forgetting—whether induced by JORs or JOLs—may be one route through which metacognitive judgments influence control decisions.

A fourth, broader account is that JOL-induced increases in restudy choices, although robust across materials, may arise from partly different processes depending on the learning context. This possibility is also consistent with the restudy–accuracy analyses: the difference in the restudy–accuracy association between the JOL and no-JOL conditions was significant for the easier related word pairs in Experiment 3, but not for the more difficult materials in Experiments 1 and 2. For unrelated word pairs, increased restudy choices may partly reflect compensatory responses to disrupted or effortful encoding. For general knowledge questions, they may reflect heightened attention to whether an answer had been sufficiently retrieved or consolidated. Related word pairs may reflect attempts to strengthen items that felt learnable but not yet fully secure. On this view, the cross-material stability of the overt effect does not necessarily imply a unitary mechanism. Instead, the same behavioral outcome—increased restudy choices—may be generated by different control processes across different types of materials.

These four accounts are not mutually exclusive. The compensatory account, the conservatism shift account, the forgetting-salience account, and the material-dependent account each capture different aspects of the data, and multiple processes may operate in concert. Because direct investigations of control-level JOL reactivity remain scarce, future research is needed to systematically test these accounts, for example, by combining restudy-choice measures with direct assessments of strategy use, decision processes, and actual restudy behavior.

### 6.2. Limitations and Future Directions

Although the present study provides new evidence for the reactivity effect of making JOLs at the level of metacognitive control, several issues need further investigation. First, participants’ restudy choices were not honored, following a procedure conceptually similar to prior work (e.g., Tullis & Benjamin, 2012). This design was useful for isolating restudy choices as an index of metacognitive control, but it reduces ecological validity because, in real learning contexts, restudy decisions typically determine subsequent study opportunities. Future research should therefore examine whether the same reactivity effect emerges when learners’ restudy choices have direct consequences for later study and memory performance (Finn, 2008; Metcalfe & Finn, 2013).

Second, although the present results were consistent across unrelated word pairs, general knowledge questions, and related word pairs, the range of tasks, materials, and participant populations was still limited. Accordingly, it would be premature to conclude that JOL-induced restudy-choice reactivity is broadly general across all learning contexts. Future studies should test the stability, scope, and boundary conditions of this effect across a wider range of paradigms and populations.

Third, previous theory suggests that metacognitive monitoring and metacognitive control may influence one another rather than operating in a strictly one-way sequence (Dunlosky & Tauber, 2016; Fleming & Daw, 2017; Nelson & Narens, 1994). Although the present study examined whether making JOLs reactively altered restudy choices, it did not test whether the act of making restudy decisions may itself exert reactive effects on later memory performance. For example, once participants indicated that an item should be restudied, they may have disengaged from that item or reduced further internal maintenance because they expected to encounter it again later. Such disengagement could influence subsequent recall performance independently of the effect of making JOLs. Future work should therefore examine whether control decisions can, in turn, influence memory in a reactive manner.

### 6.3. Methodological Implications

The present findings have implications for both JOL reactivity research and research on metacognitive control. Most importantly, they suggest that JOL reactivity cannot be understood solely in terms of memory outcomes, because making JOLs may also alter later regulation decisions. The results further indicate that future research should distinguish more clearly between memory-level and control-level reactivity. Although the present study did not directly test the mechanisms underlying this pattern, the findings are consistent with at least a partial dissociation between these two forms of reactivity. Methodologically, the present results suggest that in paradigms involving later regulation decisions, JOLs should not be treated as purely passive measures of monitoring, because making JOLs may itself alter the control behavior being measured.

## 7. Conclusions

The present study provides evidence that making JOLs can produce a reactivity effect at the level of metacognitive control, reflected in an increased tendency to select items for restudy. Across the materials examined here, this control-level effect appeared more stable than the effects of making JOLs on memory performance. More broadly, the findings suggest that JOL reactivity should be considered not only in relation to memory performance but also in relation to metacognitive control decisions.

## Data Availability

The data presented in this study are available on request from the corresponding author due to privacy.

## Note

1 If participants failed to provide a JOL within the 5 s time window, the JOL value for that trial was coded as missing. Because restudy choices in these trials were conceptually dependent on the availability of a valid JOL, the corresponding restudy data were also coded as missing and excluded from analyses of restudy proportions. Importantly, test (recall) performance data from these trials were retained and included in the analyses, as test performance was not affected by whether a JOL had been provided.

## Appendix A. Trial-Level Analyses of Restudy Choices and Subsequent Recall Accuracy

To examine whether making JOLs changed the threshold used to select items for restudy, additional trial-level mixed-effects logistic regression analyses were conducted for each experiment. Recall accuracy on each item was entered as the binary outcome variable, with condition, restudy choice, and their interaction as fixed effects. Condition (JOL vs. no-JOL) was coded with the no-JOL condition as the reference level, and restudy choice was coded as 0 = not selected for restudy and 1 = selected for restudy. Each model included random intercepts for participants and items. These analyses tested whether the relation between restudy decisions and subsequent recall accuracy differed between the JOL and no-JOL conditions. If making JOLs produced a more conservative threshold for selecting items for restudy, items selected for restudy in the JOL condition should show higher subsequent recall accuracy than items selected for restudy in the no-JOL condition, resulting in a reliable condition × restudy choice interaction. We used this model-based approach as a trial-level analogue of comparing subsequent recall performance across the four combinations of condition and restudy choice, while accounting for the non-independence of observations within participants and items.

Across the three experiments, items selected for restudy were less likely to be recalled than items not selected for restudy. This restudy-choice effect was significant in Experiment 1, *b* = −0.77, *SE* = 0.16, *z* = −4.78, *p* < .001, *OR* = 0.46, 95% CI [0.34, 0.64]; Experiment 2, *b* = −1.03, *SE* = 0.18, *z* = −5.66, *p* < .001, *OR* = 0.36, 95% CI [0.25, 0.51]; and Experiment 3, *b* = −0.45, *SE* = 0.10, *z* = −4.68, *p* < .001, *OR* = 0.64, 95% CI [0.53, 0.77]. Thus, restudy decisions were generally directed toward items that were less likely to be recalled, indicating that learners’ restudy choices were sensitive to item difficulty. The main effect of condition mirrored the memory-reactivity pattern observed in the primary analyses: recall accuracy was lower in the JOL condition than in the no-JOL condition in Experiment 1, *b* = −0.30, *SE* = 0.14, *z* = −2.17, *p* = .030, *OR* = 0.74, 95% CI [0.57, 0.97]; did not differ reliably between conditions in Experiment 2, *b* = −0.33, *SE* = 0.30, *z* = −1.09, *p* = .277, *OR* = 0.72, 95% CI [0.40, 1.30]; and was higher in the JOL condition than in the no-JOL condition in Experiment 3, *b* = 0.19, *SE* = 0.08, *z* = 2.50, *p* = .012, *OR* = 1.21, 95% CI [1.04, 1.41]. Critically, however, the condition × restudy choice interaction was not significant in any experiment: Experiment 1, *b* = 0.24, *SE* = 0.21, *z* = 1.14, *p* = .256, OR = 1.28, 95% CI [0.84, 1.94]; Experiment 2, *b* = 0.40, *SE* = 0.25, *z* = 1.64, *p* = .101, *OR* = 1.50, 95% CI [0.92, 2.43]; and Experiment 3, *b* = −0.02, *SE* = 0.13, *z* = −0.19, *p* = .846, OR = 0.98, 95% CI [0.76, 1.25]. These results indicate that restudy choices were generally sensitive to item difficulty, but they do not provide clear evidence that making JOLs reliably changed the relation between restudy decisions and later recall accuracy. Thus, although a conservative account remains a possible interpretation of the overall increase in restudy choices, the present analyses do not provide definitive support for this specific mechanism.

## Appendix B. Carryover Effects Across Blocks

To examine potential carryover effects across blocks, we fitted generalized linear mixed-effects models with a binomial logit link. Trial-level accuracy and restudy choices were predicted from the current condition, block, and within-block trial order, with random intercepts for participants. We then added the previous block’s condition (prev_cond) as a lagged predictor. When possible, we additionally tested the interaction between the current condition and prev_cond.

### Appendix B.1. Experiment 1

For Experiment 1, adding prev_cond did not improve model fit for either accuracy, *χ*^2^(1) = 1.02, *p* = .311, or restudy choices, *χ*^2^(1) = 0.19, *p* = .664. When the current condition × prev_cond interaction was added, the fixed-effect design matrix was rank-deficient, and one coefficient was dropped. As a result, the interaction term could not be reliably estimated, and the interaction model was effectively identical to the main-effects model. Overall, these analyses provided no evidence that the previous block’s condition carried over to influence accuracy or restudy choices in the subsequent block.

### Appendix B.2. Experiment 2

For Experiment 2, adding prev_cond did not improve model fit for either accuracy, *χ*^2^(1) = 0.07, *p* = .792, or restudy choices, *χ*^2^(1) = 0.48, *p* = .487. In addition, the current condition × prev_cond interaction was not significant for either accuracy, *χ*^2^(1) = 1.19, *p* = .276, or restudy choices, *χ*^2^(1) = 0.00, *p* = .979. Thus, there was no evidence that the previous block’s condition carried over to affect outcomes in the subsequent block.

### Appendix B.3. Experiment 3

For Experiment 3, adding prev_cond did not improve model fit for either restudy choices, *χ*^2^(1) = 0.09, *p* = .759, or accuracy, *χ*^2^(1) = 0.33, *p* = .563. The current condition × prev_cond interaction was also not significant for either restudy choices, *χ*^2^(1) = 0.51, *p* = .477, or accuracy, *χ*^2^(1) = 0.51, *p* = .476. Thus, there was no evidence that the previous block’s condition carried over to affect outcomes in the subsequent block.

## Appendix C. Transparency and Openness Statement

The present study was not preregistered. We report how the sample size was determined, all experimental conditions, all measures, and all data exclusion rules. No participants were excluded from the primary analyses in Experiments 1–3. For secondary gamma-correlation analyses, participants were excluded only when the relevant gamma correlation could not be computed because of insufficient variability in restudy choices.
